# Diagnostic Performance of the MeMed BV Test to Distinguish Between Bacterial and Viral or Other Non-Bacterial Causes Amongst ED and Urgent Care Patients: A Systematic Review with Meta-Analysis

**DOI:** 10.3390/diagnostics16121930

**Published:** 2026-06-22

**Authors:** Sandeep Moola, Enitan D. Carrol, Richard Rothman, Hasik PN, Andrey Maslov, Oleg Borisenko

**Affiliations:** 1 MTRC HEOR, Leeds LS12 6LN, UK; 2 Faculty of Health and Life Sciences, University of Liverpool, Liverpool L69 7ZX, UK; 3 Department of Medicine, The Johns Hopkins University, Baltimore, MD 21218, USA

**Keywords:** MeMed BV, ImmunoXpert™, host-response, bacterial infection, viral infection, respiratory tract infection, fever, diagnostic accuracy, systematic review, meta-analysis

## Abstract

**Background/Objectives**: Respiratory tract symptoms, urinary symptoms, and acute fevers frequently prompt emergency urgent care visits. Distinguishing bacterial from viral or non-bacterial etiology remains difficult because clinical features overlap and laboratory microbiological tests are often non-specific or delayed. The MeMed BV^®^ test is a rapid host-response assay that combines TRAIL, IP-10, and CRP into a composite score to differentiate between bacterial and viral/non-bacterial infections within 15 min. The objective of this systematic review and meta-analysis was to evaluate the diagnostic accuracy and clinical utility of the MeMed BV test in adults and children with suspected respiratory tract infections, urinary tract infections, and undifferentiated fever. **Methods**: The review followed PRISMA-DTA guidelines. Medline, Embase, CINAHL, and the Cochrane Library databases were searched. The risk of bias was assessed using the QUADAS-2, Cochrane RoB 2.0, ROBINS-I, and JBI tools. Where appropriate, meta-analyses were performed using a bivariate random-effects or HSROC model. **Results**: Sixteen studies (12 diagnostic test accuracy (DTA) studies and four non-DTA studies) were included. The pooled sensitivity was 91% (95% CI: 86–94%), and specificity was 92% (95% CI: 91–93%), with consistent accuracy in adults (Sensitivity 93%/Specificity 91%) and children (Sensitivity 88%/Specificity 93%). The non-DTA studies demonstrated that MeMed BV-guided management improved antibiotic stewardship: antibiotics were prescribed in 20.6% of viral versus 73.2% of bacterial cases, and clinician adherence to MeMed BV results reached 75–80%. **Conclusions**: The MeMed BV test demonstrates consistently high diagnostic accuracy and is associated with improved antibiotic decision-making, supporting its integration into clinical workflows.

## 1. Introduction

Respiratory tract infections (RTIs), urinary tract infections (UTIs), and undifferentiated fever are among the most common clinical presentations in emergency and urgent care settings. In the United States, nationally representative ED data show that diseases of the respiratory system account for 8.9% of ED visits and diseases of the genitourinary system for 4.7%; UTI with site not specified and undifferentiated fever each account for around 1% of ED visits (1.1% and 1.2%, respectively) [1]. In European emergency departments, fever is also one of the most frequent presenting symptoms in pediatric populations, accounting for approximately 20% of pediatric ED visits [2]. During each of these encounters, an acute care healthcare provider will decide whether to prescribe antibiotics based on their clinical impression, coupled with limited diagnostic tools, to help determine whether the observed syndrome(s) reflect the patient’s response to a bacterial infection or another infection type. However, it is well established that infections of bacterial and viral etiology exhibit overlapping clinical presentations and are often difficult to differentiate.

Diagnostic uncertainty is a key challenge in clinical decision-making and a recognized driver of inappropriate antibiotic prescribing [3], and as a result, antibiotics are among the most misused drugs in the world [4]. Public health organizations estimate that 30–50% of antibiotic prescriptions are unnecessary; further, antibiotic prescriptions are inappropriately delayed in approximately 20% of cases, with important potential downstream adverse complications [4,5]. In Europe, studies suggest that more than 50% of antibiotics prescribed for RTIs, the most common infectious syndrome, were inappropriate [6,7,8]. Antibiotic misuse is directly linked to poor patient outcomes, [9] and the increasing worldwide burden of antimicrobial resistance [10,11]. Antimicrobial resistance was responsible for an estimated 1.27 million deaths globally and accounted for approximately 4.95 million deaths in 2019, reflecting both deaths directly attributable to resistance and those in which resistance was a contributing factor [10]. Among the various strategies experts recommend to curtail global antimicrobial resistance, developing new, reliable, rapid diagnostic tools is amongst the most important to help guide clinicians in making more informed treatment decisions that optimize appropriate antibiotic use.

The current, most well-established laboratory tests commonly available to acute care clinicians assessing a patient with a suspected infection have significant shortfalls in providing clarity about whether the causative pathogen is bacterial or nonbacterial. Standard microbiological tests, such as culture and RT-PCR, can detect specific pathogens. However, well-documented challenges exist with these tests, including but not limited to the inability to distinguish pathogens from colonizers, difficulty in reliably accessing the infection site, poor performance of these tests for emerging pathogens, and long processing times (i.e., with culture), limiting the overall utility of these traditional tools when making antibiotic prescribing decisions. Further, even though emerging rapid viral testing platforms have been developed, global access to these technologies is frequently limited, the number of pathogen targets is limited, and these tests cannot identify potential bacterial co-infections. Other common laboratory tests that measure physiological responses, such as changes in white blood cell populations and inflammatory markers (e.g., C-reactive protein or procalcitonin), are nonspecific. None of the laboratory tests described above was designed specifically to distinguish whether an observed clinical syndrome is most likely caused by a bacterial pathogen and warrants antibiotic treatment.

The MeMed BV test was specifically designed to differentiate between bacterial and non-bacterial etiology in patients presenting with infectious syndromes [12]. The MeMed BV test measures blood levels of three host proteins: tumor necrosis factor-related apoptosis-inducing ligand (TRAIL), interferon gamma-induced protein 10 (IP-10), and C-reactive protein (CRP) to create a composite score that indicates the likelihood of a host immune response to a bacterial or viral infection [12,13,14]. Each of these proteins is associated with distinct immune pathways activated in response to bacterial or viral infection. CRP elevates as part of the inflammatory response, but this elevation is more pronounced in response to bacterial infection. IP-10 upregulates during any infection, but the elevation is more pronounced during viral infections. TRAIL upregulates during viral immune response but downregulates during bacterial immune response [12,15,16,17,18]. The concentrations of these biomarkers are computationally integrated into a score ranging from 0 to 100, with predefined thresholds corresponding to viral/non-bacterial, equivocal, or bacterial response patterns [12,19]. By integrating biomarkers reflecting distinct host immune-response pathways into a single score, the test is intended to support differentiation between bacterial and viral/non-bacterial infections when clinical presentation and routine laboratory findings overlap or remain inconclusive. Initially launched as a manual ELISA-based kit under the name ImmunoXpert, the test has now been advanced to an automated format on the MeMed Key platform, delivering results within 15 min [20]. Both the MeMed BV test and the ImmunoXpert test use the same biomarker signature and are analytically equivalent [21].

Despite increasing adoption, the overall diagnostic accuracy of the MeMed BV test across various clinical indications has not as of yet been fully characterized, particularly for those patients most commonly encountered in acute care settings, namely those with suspected RTIs, UTIs, or undifferentiated fever. Variations in study designs, population groups, reference standards, and settings limit the interpretation of test performance. This limits the generalizability of individual study findings and underscores the need for a comprehensive synthesis, including a meta-analysis.

A recent review of PROSPERO (last search 23 June 2025) identified only three related records: one focused on febrile children (CRD42024506430), one on novel point-of-care tests (CRD42020178973), and one registered under CRD42023427097, corresponding to the UK NIHR-commissioned systematic review of reviews by Webster et al. (2024) [22]. The NIHR review was broad in scope, covering multiple host-response assays and various clinical contexts, but did not conduct a focused quantitative synthesis of MeMed BV evidence for acute care use.

To our knowledge, this is the first systematic review and meta-analysis to focus exclusively on the diagnostic accuracy and clinical utility of the MeMed BV test, including studies conducted in pediatric and adult populations in emergency and urgent care settings. The focus on RTIs, UTIs, and undifferentiated fever was intentional and pre-specified in the review protocol and reflects the intended clinical use of the MeMed BV test in emergency and urgent care settings. The review assessed the diagnostic performance of the MeMed BV test in distinguishing between bacterial and viral/non-bacterial infections and evaluated its clinical utility in improving patient outcomes and supporting antimicrobial stewardship in emergency and urgent care settings.

## 2. Materials and Methods

### 2.1. Protocol and Registration

The protocol for this systematic review was prospectively registered with PROSPERO (CRD420251112437). The protocol adhered to the PRISMA-DTA guidelines (A completed PRISMA 2020 checklist is provided as Supplementary Materials), ensuring methodological rigor and transparency in the assessment of diagnostic test accuracy.

### 2.2. Eligibility Criteria

Studies were eligible for inclusion if they met the specified criteria related to population, index test, comparators, reference standard, outcomes, and study design. These criteria were established a priori to ensure the relevance and methodological rigor of the included evidence.

#### 2.2.1. Population

The systematic review included studies involving adults (≥18 years) or children (≥90 days to 18 years) presenting to emergency departments and urgent care settings with suspected RTIs, UTIs, or undifferentiated fever, as defined in the individual studies, generally based on measured or reported fever thresholds (≥37.5 °C). Studies with mixed settings (ED and hospital inpatient) were included only if more than 50% of the patients were recruited from the ED or urgent care setting, and the infection was not already confirmed at the time of recruitment.

#### 2.2.2. Index Test

The review focused on evaluating the diagnostic performance of the MeMed BV and ImmunoXpert tests (same protein targets and scoring algorithm with equivalent performance [21], designed to differentiate patients presenting with bacterial infection (or bacterial co-infection) from patients presenting with a viral/non-bacterial infection. These tests analyze the expression levels of three key immune biomarkers: TRAIL, IP-10, and CRP, which are translated into a composite score for interpretation.

#### 2.2.3. Reference Standards

Reference standards for establishing the most likely infection etiology (i.e., bacterial vs. viral) included standard diagnostic methods, such as clinical adjudication (based on laboratory and imaging studies) and/or microbiological testing (e.g., cultures, PCR).

#### 2.2.4. Comparators

Comparators reflected routine diagnostic practice and included commonly used biomarkers (e.g., CRP, procalcitonin (PCT), WBC, ANC), microbiological tests such as cultures and PCR panels, clinical judgment or standard of care, and predefined diagnostic algorithms that combined laboratory, microbiology, and imaging assessments.

#### 2.2.5. Outcomes

The primary outcome included diagnostic accuracy metrics (sensitivity, specificity, predictive values, AUC, and likelihood ratios). Secondary outcomes included impact on patient outcomes and antibiotic prescribing, diagnostic error reduction, patient management, time to diagnosis, and workflow integration. The review excluded studies that reported health economic outcomes (cost, cost-effectiveness, budget impact analysis).

#### 2.2.6. Study Designs

The review included diagnostic test accuracy (DTA) studies designed to evaluate the diagnostic performance of the MeMed BV or ImmunoXpert test. Relevant randomized controlled trials, non-randomized controlled trials, cohort studies, and before-and-after studies were also included.

### 2.3. Information Sources and Search Strategy

A comprehensive literature search was conducted across the following electronic databases: Medline (including Medline In-Process), Embase, CINAHL, and the Cochrane Library. Searches were performed on 02 July 2025. In addition, the reference lists of all relevant systematic literature reviews identified during screening were examined to identify any additional eligible studies not captured through the database searches. The search strategy was developed using a combination of MeSH terms and keywords relevant to the index test and related diagnostic technologies.

No restrictions were placed on the publication date, allowing for the inclusion of studies published at any time. Only full-text, peer-reviewed publications in the English language were considered. Appendix A provides detailed search strategies for four databases.

### 2.4. Study Selection and Screening

**Figure 1:**
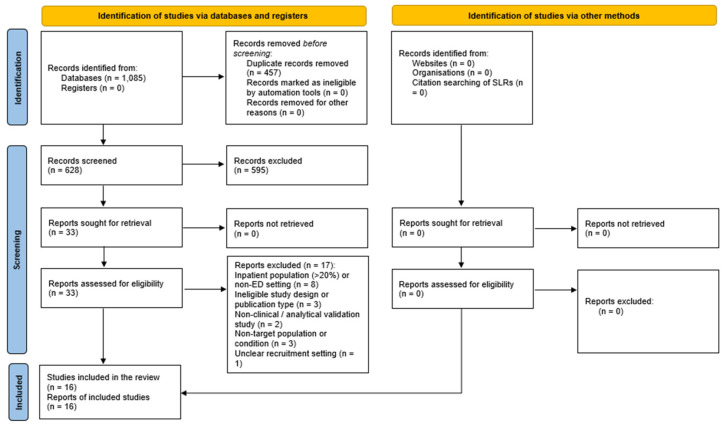
PRISMA flowchart (Page 2021).

Two reviewers independently screened titles/abstracts and full texts, resolving disagreements by consensus or through a third reviewer. Reasons for exclusion at the full-text stage were documented. The study selection process is presented in a PRISMA flow diagram (Figure 1).

### 2.5. Data Extraction

A standardized template was piloted and applied to all included studies, capturing study design, setting, population, index test, reference standard, outcomes, and funding source. One reviewer extracted the data, and a secondary reviewer verified all data.

A standardized data extraction form was used to collect key information from each included study, which contained study characteristics (author, year, country, design, setting, sample size), population details (age, sex, inclusion/exclusion criteria, and age group), target condition (defined as the primary suspected clinical syndrome at presentation, as specified by the study authors and pre-specified enrolment criteria: upper or lower respiratory tract infection, urinary tract infection, or undifferentiated fever), index test details (MeMed BV platform, interpretation, and cut-off values), and reference standards (e.g., microbiological culture, RT-PCR, or expert clinical adjudication).

Extracted outcomes, where reported, included diagnostic performance metrics (sensitivity, specificity, true/false positives and negatives, predictive values, likelihood ratios, and AUC), as well as secondary measures such as time to result, impact on clinical decisions, antibiotic prescribing, patient outcomes, and test failures. Key results and funding sources were also recorded.

### 2.6. Risk of Bias Assessment

Two reviewers assessed the risk of bias (RoB) using validated tools: QUADAS-2 for DTA studies [23], Cochrane RoB 2.0 for randomized controlled trials [24], and the JBI checklist for cohort studies [25]. For non-comparative observational decision-impact studies evaluating the effect of MeMed BV on clinical decision-making, risk of bias was assessed using the ROBINS-I tool for non-randomized studies of interventions [26]. All risk-of-bias assessments were reported in tabular format, with domain-level judgments and supporting justifications provided for each domain rating.

### 2.7. Data Synthesis

Where appropriate, meta-analysis was conducted to estimate the pooled diagnostic accuracy and clinical effectiveness of the MeMed BV test. When ≥80% of studies reported accuracy at the manufacturer’s fixed cut-off (viral = 0–34, equivocal = 35–65, bacterial = 66–100), logit-transformed sensitivity and specificity were pooled in a bivariate REML random-effects model, accounting for within-study correlation and yielding pooled estimates with 95% CIs and a 95% prediction ellipse. If >20% of studies reported varying thresholds, a hierarchical summary ROC (HSROC) model was used. A hierarchical summary ROC (HSROC) model was prespecified to examine potential threshold effects and between-study heterogeneity. Given that all the included studies applied uniform manufacturer thresholds, the primary analyses were based on the bivariate model, with HSROC analyses presented as supportive assessments of diagnostic performance. Analyses were performed in Stata (midas, metandi), with ROC plots generated in Stata v19.

Where studies reported results using the standard MeMed BV score thresholds (viral/non-bacterial: 0–34; equivocal: 35–65; bacterial/co-infection: 66–100), a common threshold was applied across studies. Between-study variance was assessed in accordance with Cochrane DTA guidance. Meta-analyses were complemented by a narrative synthesis, supported by tables and figures that presented key diagnostic metrics. Subgroup or meta-regression analyses were conducted, where feasible, by patient population (children vs. adults) and risk of bias. Publication bias was formally assessed using Deeks’ funnel plot asymmetry test [27]. Conventional funnel plots were also generated as exploratory visual assessments.

## 3. Results

A comprehensive search across four databases yielded 1085 records. Following the removal of 457 duplicates, 628 unique records were screened by title and abstract against the inclusion and exclusion criteria specified in the protocol. Of these, 595 records were excluded because they did not meet the predefined eligibility criteria. The remaining 33 studies were retrieved for full-text review, and following a detailed eligibility assessment, 17 studies were excluded. As shown in Figure 1, supplementary search methods, including manual screening of the reference lists of relevant systematic reviews, did not identify any additional eligible studies. A total of 16 publications were finally included in the review. Figure 1 shows the PRISMA study selection flowchart.

The literature search identified 16 eligible studies evaluating the MeMed BV test (also known as ImmunoXpert) for differentiating between bacterial and viral/non-bacterial infections in acute-care settings. Of these, 12 were DTA studies, providing sufficient data for quantitative synthesis, while four were non-DTA studies reporting on outcomes such as antibiotic prescribing, clinical decision-making, or analytical performance. Several studies enrolled patients with mixed infectious syndromes and did not consistently report extractable syndrome-specific diagnostic accuracy data (RTIs, UTIs, and fever). Therefore, pooled analyses were conducted by age group rather than by clinical presentation. Table 1 and Table 2 summarize the included studies and detail their key characteristics.

Appendix B provides a list of the 17 studies that were excluded, along with the reasons for their exclusion. The studies were excluded mainly because they were inpatient-only populations, non-relevant settings, ineligible publication types, focused on narrow subgroups or pathogens, lacked formal diagnostic accuracy analyses, or were unclear about the distribution of recruitment between emergency/urgent and inpatient care.

### 3.1. Study Characteristics

A total of 16 studies met the inclusion criteria, comprising 12 DTA studies [12,13,17,20,28,29,30,31,32,33,34,35], and four non-DTA studies [19,36,37,38]. The DTA studies primarily assessed the diagnostic performance of the MeMed BV test across diverse populations and settings. The non-DTA studies provided complementary evidence on clinical utility, prescribing behavior, and workflow integration. An additional non-DTA study by Kalmovich et al. (2026), published after completion of the primary literature searches, was identified during manuscript drafting and included because of its relevance to clinical utility outcomes [39]. The study underwent the same eligibility assessment and data extraction procedures as the other included non-DTA studies [39].

The 12 DTA studies were published between 2015 and 2024 and together included 6359 participants in their primary analyses. These studies were conducted in Israel, the United States, Spain, Italy, Germany, Switzerland, and the UK. Most studies were prospective and cross-sectional DTA studies. Across all studies, the number of included patients varied: the total number of enrolled patients ranged from 60 to 3003 (median: 511; IQR: 442–1006), while the number of patients included in the final analyses ranged from 60 to 1561 (median: 388; IQR: 280–826).

Several studies initially enrolled larger cohorts of patients but excluded those with indeterminate reference diagnoses or uncertain etiologies, meaning the adjudication process did not yield a conclusive etiological assignment and, therefore, the cases could not serve as a reference standard for diagnostic accuracy calculations. In some studies, additional exclusions occurred because patients did not meet predefined eligibility criteria or had insufficient clinical or laboratory information available for etiological classification. These exclusions did not necessarily indicate non-infectious disease, but commonly reflected uncertainty regarding definitive bacterial or viral classification, insufficient clinical information, or inability to meet predefined study criteria. For example, Klein et al. (2023) enrolled 3003 patients but analyzed only 1561 (848 were determined ineligible after enrollment, and 594 could not be assigned to a reference standard etiology) [32]. Similarly, Srugo et al. (2017) enrolled 529 patients but analyzed only 307 (168 were determined ineligible after enrollment, and 54 could not be assigned to a reference standard) [13]. All studies were conducted in emergency departments and/or urgent-care centers and included patients presenting with suspected RTI, undifferentiated fever (particularly in children), or, less commonly, UTI. Some studies enrolled both adults and children, with results usually reported separately, allowing stratified meta-analysis.

The index test was either the manual ELISA-based ImmunoXpert, most commonly used in earlier studies [12,13,17,29,32], or the automated MeMed Key platform used in more recent multicenter trials [20,28,31]. Both platforms measure the same host-response signature (TRAIL, IP-10, and CRP), calculate the same composite score, and provide the same interpretation thresholds: viral (0–34), equivocal (35–65), or bacterial (66–100). The derivation study by Oved et al. (2015) used different index test cut-offs (0.35 and 0.55), whereas all other studies used the manufacturer thresholds of 0.35 (lower) and 0.65 (higher) [12].

Reference standards varied, including microbiological confirmation (culture, PCR), expert panel adjudication, or hybrid definitions that combined laboratory, imaging, and clinical data. The majority of included studies relied on adjudication by independent infectious disease and emergency medicine experts, who were blinded to BV results and informed by the clinical presentation and available diagnostic data. Some studies included follow-up periods of up to 30 days to confirm diagnoses [17,30].

Comparators included procalcitonin (PCT), CRP, and clinician judgment. In almost all DTA analyses, indeterminate reference-standard classifications or equivocal composite scores were excluded from sensitivity, specificity, NPV, and PPV calculations. Performance analyses that reported the area under the receiver operating curve (AUC) and bin-based performance tables incorporated equivocal MeMed BV results. Funding sources were mixed, including both industry-sponsored and investigator-initiated studies.

The five non-DTA studies provided supplemental evidence on clinical utility outcomes. Four of the five studies were conducted between 2023 and 2025. Fröhlich et al. (2023) conducted a prospective cohort study in Germany [36], enrolling 53 children and adolescents with suspected infections. Kalmovich et al. (2023) and Kalmovich et al. (2025) were prospective, non-comparative studies conducted in Israel, in which clinicians recorded their likelihood to prescribe antibiotics in a survey provided to clinicians when the MeMed BV test was ordered, as well as actual prescribing decisions and perceived test impact, assessed after results were disclosed during the clinical encounter [37,38]. Kalmovich et al. (2023) included 152 patients [37], and Kalmovich et al. (2025) conducted a large cohort study analyzing 3758 adult patients across multiple urgent care settings [38]. Kalmovich et al. (2026) published a large prospective cohort study evaluating real-world use of the MeMed BV host-protein test in 2016 pediatric patients presenting to 10 urgent care centers in Israel [39]. Singer et al. (2025) conducted a planned feasibility randomized controlled trial that enrolled 214 adults presenting with suspected lower RTIs in the United States [19].

### 3.2. Risk of Bias Assessment of Diagnostic Test Accuracy Studies

**Figure 2:**
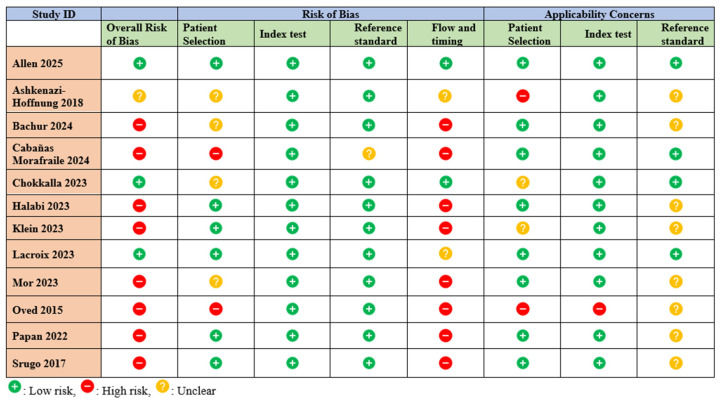
QUADAS-2 risk of bias assessment summary by domains. (Allen 2025, Ashkenazi-Hoffnung 2018, Bachur 2024, Cabañas Morafraile 2024, Chokkalla 2023, Halabi 2023, Klein 2023, Lacroix 2023, Mor 2023, Oved 2015, Papan 2022, Srugo 2017) [12,13,17,20,28,29,30,31,32,33,34,35].

The risk of bias assessment using the QUADAS-2 tool [23] indicated that the methodological quality of most studies was acceptable across key domains, although several important limitations were noted (Figure 2). Eligibility criteria were generally well-defined, the MeMed BV test was applied according to the manufacturer’s instructions, and reference standards in the majority of cases involved blinded expert adjudication. Accordingly, the index test and reference standard domains were generally rated as having a low risk of bias, with minimal concerns about applicability.

The main area of concern was the flow and timing domain. In all studies, except for three [28,31,33], patients with indeterminate reference diagnoses and/or MeMed BV scores in the equivocal (non-actionable) range (35–65) were excluded from sensitivity, specificity, NPV, and PPV calculations. In several studies, exclusions were not clearly justified, or patient clinical workflow (e.g., timing of testing, adjudication processes, and handling of uncertain cases) was insufficiently reported. Inadequate reporting may result in inconsistent patient selection and analysis across studies. Such exclusions may lead to overestimation of diagnostic performance, particularly when indeterminate reference standard results reflect underlying diagnostic uncertainty. Therefore, the flow and timing domain in the QUADAS-2 tool was rated as high risk of bias in the majority of the included studies. In contrast, Allen et al. (2025) [28], Chokkalla et al. (2023) [31], and Lacroix et al. (2023) [33] clearly reported their inclusion criteria and the handling of equivocal cases, and were therefore rated as low risk of bias.

The patient selection domain was the primary source of variability. While some studies, such as Allen et al. (2025) [28], Klein et al. (2023) [32], Lacroix et al. (2023) [33], and Srugo et al. (2017) [13] clearly described consecutive or prospective enrollment, several other studies (Bachur et al. (2024), Mor et al. (2023), Ashkenazi-Hoffnung et al. (2018)) [20,29,34] lacked a detailed description of the recruitment methods, leading to unclear ratings. Other studies (Oved et al. (2015), Cabañas Morafraile et al. (2024)) [12,30] were considered high-risk due to non-consecutive or convenience sampling. Recruitment outside routine working hours was not explicitly reported in either study; however, Cabañas Morafraile et al. (2024) [30] relied on investigator-availability-based sampling, whereas Oved et al. (2015) [12] did not specify the timing of recruitment. The index test was consistently judged to be of low risk across all studies, as the MeMed BV test was applied with manufacturer-recommended thresholds and interpreted in a blinded manner, independent of the reference standard. Reference standards were generally well reported, with one study (Cabañas Morafraile et al. (2024)) [30] rated unclear due to limitations in its reporting.

Regarding the overall risk of bias, Allen et al. (2025) [28], Chokkalla et al. (2023) [31], and Lacroix et al. (2023) [33] were rated as having a low risk across all domains, reflecting a robust methodological design, prospective enrollment, and clear blinding procedures. One study (Ashkenazi-Hoffnung et al. (2018)) [29] was deemed unclear, primarily due to the incomplete reporting of recruitment and follow-up processes. The remaining studies, including Bachur et al. (2024) [20], Cabañas Morafraile et al. (2024) [30], Halabi et al. (2023) [17], Klein et al. (2023) [32], Oved et al. (2015) [12], Papan et al. (2022) [35], and Srugo et al. (2017)[13], were assessed as high overall risk of bias, mainly due to the selective exclusion of patients, incomplete flow data, and/or retrospective components.

Applicability concerns were minimal for the index test and reference standard across all studies. Applicability concerns related to patient selection were identified in two studies (Ashkenazi-Hoffnung et al. (2018) [29], Oved et al. (2015) [12]), primarily because they included a substantial proportion of hospitalized patients alongside emergency department presentations.

### 3.3. Risk of Bias Assessment of Non-Diagnostic Test Accuracy Studies

The risk of bias for non-DTA studies was evaluated using tools appropriate to each study design: the Cochrane RoB 2.0 tool for randomized controlled trials, and the JBI checklist for cohort studies. Prospective non-comparative observational decision-impact studies evaluating the effect of MeMed BV on clinical decision-making were assessed using the ROBINS-I tool for non-randomized studies of interventions. Risk of bias assessment summaries are presented in Table 3. The ROBINS-I and JBI cohort critical appraisal summaries are included in Appendix C.

The prospective cohort study by Fröhlich et al. (2023) [36] was found to have some concerns regarding the risk of bias. In the study, exposure and outcomes were measured consistently and accurately, and appropriate analytical methods were employed. However, the main concerns were incomplete reporting or adjustment for potential confounders and limited detail on follow-up completeness.

The prospective, non-comparative studies by Kalmovich et al. (2023) [37], Kalmovich et al. (2025) [38], and Kalmovich et al. (2026) [39] were judged to be at serious risk of bias overall. All three studies had methodological strengths, including clear temporal sequencing: clinician intent was assessed prior to the reporting of the MeMed BV result; prescribing decisions were documented after result disclosure within the same clinical encounter; and antibiotic prescribing outcomes were reliably captured. However, the non-comparative design across the studies lacked an independent control group, and MeMed BV testing was ordered at the clinician’s discretion, introducing unadjusted confounding by indication. In addition, reliance on clinician-reported intention and perceived test impact introduced potential measurement and reporting bias. In Kalmovich et al. (2025) [38] and Kalmovich et al. (2026) [39] studies, discretionary test ordering and incomplete questionnaire data further raised concerns about selection bias and missing data bias.

### 3.4. Key Results

#### 3.4.1. Diagnostic Accuracy of MeMed BV

Of the 12 DTA studies, 10 studies were included in meta-analyses. One study by Oved et al. (2015) [12] was excluded from the meta-analysis because it used different index test cut-offs (0.35 and 0.55), and it was the derivation study for the MeMed BV signature. All other studies employed the manufacturer’s thresholds of 0.35 (lower) and 0.65 (higher). The study by Allen et al. (2025) [28] was not included in the meta-analysis as it reported pooled diagnostic performance metrics from three other studies that are included in the meta-analysis (Bachur et al. (2024) [20], Halabi et al. (2023) [17], and Papan et al. (2022) [35]). However, the findings from both excluded studies were consistent with those of the 10 included studies in the meta-analyses.

The diagnostic accuracy study by Oved et al. (2015) was not included in the meta-analysis because it represents the original derivation and initial validation of the MeMed BV host-response signature, and used different analytic cut-offs from all subsequent published studies [12]. This study evaluated the MeMed BV host-response signature derived from a mixed cohort of 765 patients (432 children, 333 adults) presenting with suspected acute bacterial or viral infections in emergency and hospital settings, reported an AUC of 0.94 (95% CI: 0.92–0.96), with a sensitivity of 87% and a specificity of 90% against an expert-adjudicated reference standard [12].

Allen et al. (2025) reported a pooled diagnostic accuracy analysis combining data from three prospective studies and used a novel adjudication-based reference standard designed to capture diagnostic uncertainty [28]. The analysis combined data from 1016 patients (584 adults and 432 children) with suspected acute bacterial or viral infections presenting to the emergency department and urgent care settings. Infection etiology was determined through a structured, multi-round expert adjudication process, resulting in four reference standard cohorts of increasing diagnostic stringency: microbiologically confirmed (n = 427), unanimous (n = 565), suspected (n = 860), and all-inclusive (n = 1016) [28].

Across all three cohorts included in the Allen et al. (2025) study, MeMed BV demonstrated high diagnostic accuracy for differentiating bacterial from viral or non-bacterial infections [28]. The area under the receiver operating characteristic curve (AUC) was 0.98 (95% CI: 0.94–1.00) in the microbiologically confirmed cohort, 0.98 (0.95–1.00) in the unanimous cohort, 0.95 (0.92–0.98) in the suspected cohort, and 0.90 (0.87–0.93) in the all-inclusive cohort. In contrast, procalcitonin showed substantially lower discrimination across all reference standards, with AUCs ranging from 0.69 to 0.77 [28].

Using manufacturer-defined cutoffs and excluding equivocal results, MeMed BV sensitivity ranged from 100% in the microbiologically confirmed cohort to 88.9% in the all-inclusive cohort, while specificity decreased from 92.0% to 82.5% as diagnostic uncertainty increased [28]. Similar performance was observed in adult and pediatric subgroups. Although diagnostic accuracy declined as increasingly difficult-to-classify cases were included, MeMed BV consistently outperformed procalcitonin across all reference standards [28].

Across the 10 studies included in the meta-analyses that include both adults and children with suspected bacterial or viral infection, the MeMed BV test demonstrated consistently high diagnostic accuracy. Table 4 presents the pooled diagnostic performance of MeMed BV from meta-analysis, showing sensitivity and specificity (with 95% CIs) for adults, children, and the overall population. Using the manufacturer’s fixed threshold and a bivariate random-effects model with unstructured covariance, the pooled sensitivity was 91% (95% CI: 86–94%), and specificity was 92% (95% CI: 91–93%). These results demonstrate a strong ability to accurately identify both bacterial and viral/non-bacterial infections across diverse clinical presentations in emergency and urgent care settings. A weak negative correlation was observed between sensitivity and specificity (ρ = −0.31), indicating minimal evidence of a threshold effect.

**Figure 3:**
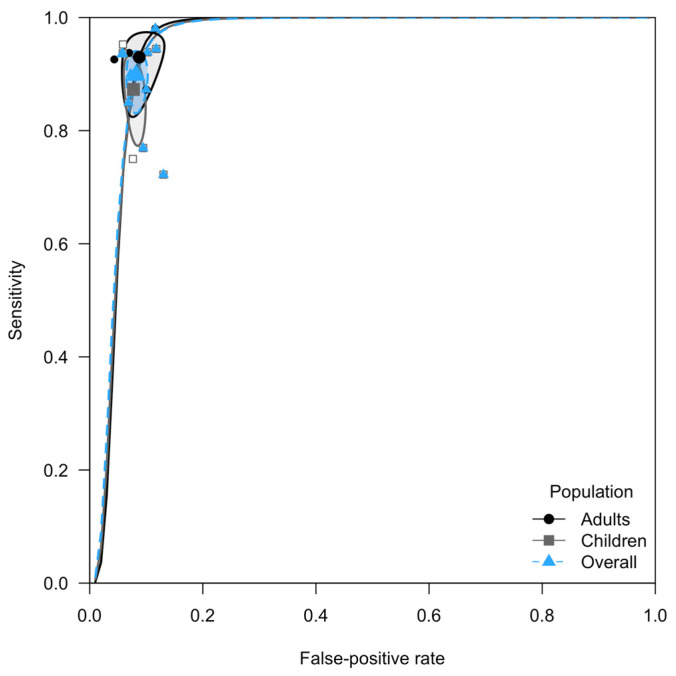
Overall pooled diagnostic accuracy of MeMed BV across adults and children: HSROC model with unstructured covariance. Circles indicate adult studies, squares denote pediatric studies, and triangles denote the overall analysis. The corresponding HSROC curves and confidence regions are shown for each population. The substantial overlap among curves and regions indicates similar diagnostic performance across adult and pediatric populations.

Figure 3 presents the diagnostic performance of adults (black circles and curve with shaded ellipse) and children (gray squares and curve with shaded ellipse), with each point representing an individual study. The shaded ellipses represent 95% confidence regions for the pooled sensitivity and specificity. The overlays for adults and children show that both populations cluster in the high-sensitivity, low-false-positive region of ROC space. The confidence regions largely overlap, indicating that the diagnostic performance of MeMed BV was broadly comparable across age groups. This suggests that the test can distinguish between bacterial and viral/non-bacterial infections with similar accuracy in both adults and children.

**Figure 4:**
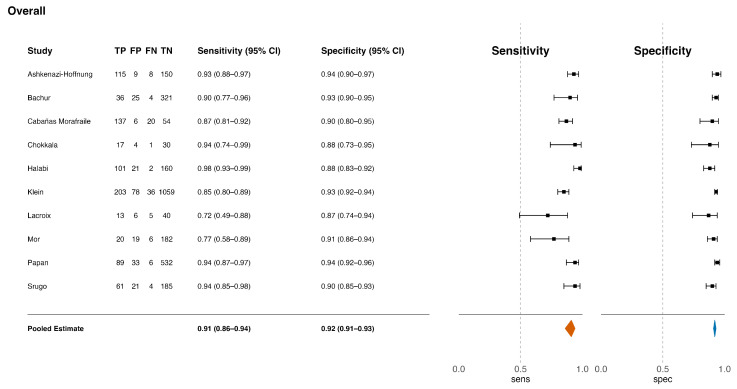
Coupled forest plot of study-level sensitivity and specificity estimates for the MeMed BV test (overall). Black squares represent individual study estimates, and horizontal lines indicate 95% confidence intervals. The orange diamond indicates the pooled sensitivity estimate, and the blue diamond denotes the pooled specificity estimate.

Figure 4 presents a paired forest plot displaying study-level estimates of sensitivity and specificity (with 95% CIs) along with pooled estimates for the MeMed BV test across all included diagnostic accuracy studies. Each horizontal line represents the 95% CI around the point estimate for an individual study. Each square represents an individual study, with horizontal lines indicating confidence intervals. Diamonds denote the pooled sensitivity and specificity estimates; the orange diamond denotes the pooled sensitivity estimate, and the blue diamond denotes the pooled specificity estimate.

Sensitivity values ranged from 0.72 to 0.98, and specificity values ranged from 0.82 to 0.94, indicating consistently high diagnostic performance across studies, with some variability. Most studies clustered toward the upper-right of the plot, reflecting the strong ability of the MeMed BV test to accurately identify both bacterial and viral infections.

Table 5 summarizes the overall diagnostic accuracy of MeMed BV across individual studies, including the total number of patients analyzed and the reported sensitivity and specificity with 95% confidence intervals. Some studies reported diagnostic performance at predefined clinical cutoffs (sensitivity/specificity) without providing AUC values. Where available, AUC values are also presented. The AUC value reflects the discriminatory performance of the continuous MeMed BV score across all thresholds and is independent of predefined viral, equivocal, and bacterial cutoffs. Sensitivity and specificity are calculated at fixed clinical thresholds and may depend on how equivocal test results and indeterminate reference-standard cases are handled.

#### 3.4.2. Diagnostic Accuracy of MeMed BV in Adults

Four studies, including around 850 adults, contributed to the pooled analysis (Ashkenazi-Hoffnung et al. (2018) [29], Bachur et al. (2024) [20], Cabañas Morafraile et al. (2024) [30], Halabi et al. (2023) [17]). At the fixed manufacturer threshold, the MeMed BV test achieved a pooled sensitivity of 93% (95% CI: 87–97%) and specificity of 91% (95% CI: 88–94%) under the unstructured covariance model.

Sensitivity analyses using an independent covariance structure produced identical estimates, supporting the robustness of the results. The HSROC model yielded a summary operating point with a sensitivity of 90.7% (95% CI: 75–97%) and a specificity of 94.2% (95% CI: 73.9–98.9%), corresponding to a diagnostic odds ratio (DOR) of 157 (95% CI: 66.7–377.9). The findings confirm the high overall discriminative ability of the test across varying decision thresholds.

**Figure 5:**
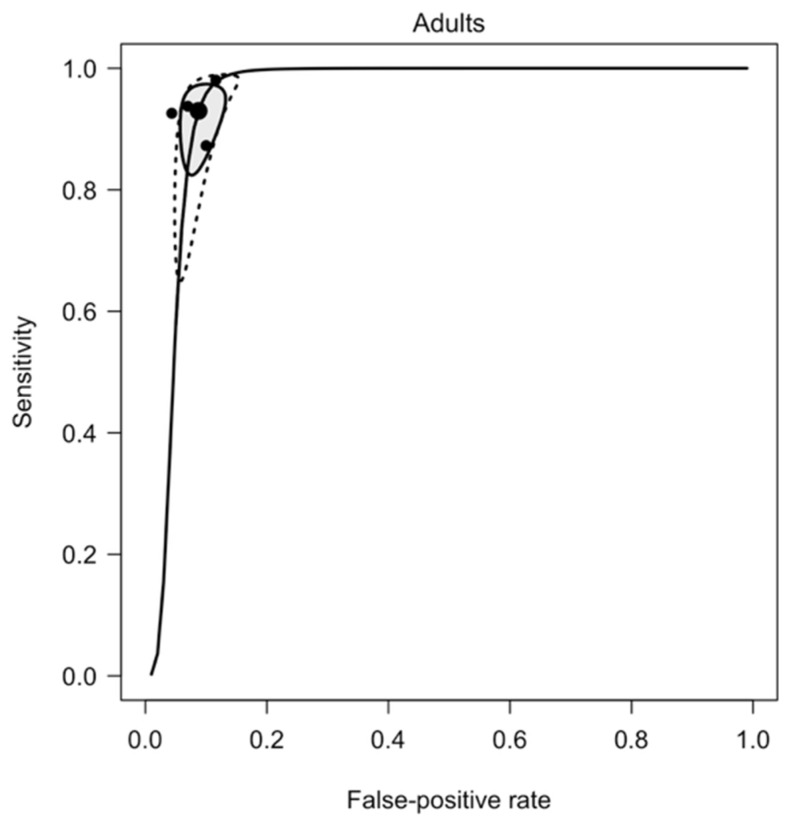
Pooled diagnostic accuracy of MeMed BV in adults: HSROC model with unstructured covariance. The solid curve represents the fitted HSROC curve. The solid ellipse indicates the 95% confidence region around the summary operating point, and the dotted ellipse denotes the 95% prediction region. The gray background represents the plotting area.

Figure 5 presents the diagnostic performance in adults, with black circles representing individual studies and the solid black curve showing the summary ROC. The studies cluster in the high-sensitivity, low-false-positive region, and the shaded confidence region around the curve indicates consistently strong and reliable test performance for distinguishing bacterial from viral/non-bacterial infections in adults.

**Figure 6:**
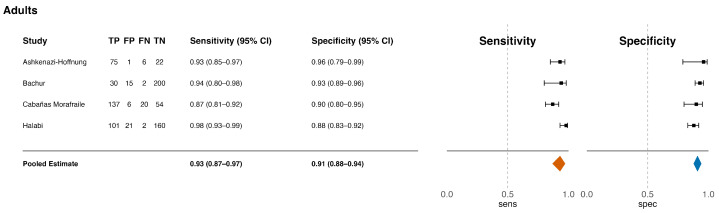
Coupled forest plot of study-level sensitivity and specificity estimates for the MeMed BV test (adults). Black squares represent individual study estimates, and horizontal lines indicate 95% confidence intervals. The orange diamond indicates the pooled sensitivity estimate, and the blue diamond denotes the pooled specificity estimate.

Figure 6 presents a coupled forest plot showing study-level estimates of sensitivity and specificity (with 95% CIs) for the MeMed BV test among adult participants across the included diagnostic accuracy studies. Sensitivity values ranged from 0.87 to 0.98, and specificity ranged from 0.88 to 0.96, demonstrating consistently high diagnostic performance across studies, with limited between-study variability.

#### 3.4.3. Diagnostic Accuracy of MeMed BV in Children

Eight pediatric studies (generally including children aged 3 months to 18 years) were pooled. Using a bivariate random-effects model with unstructured covariance, the MeMed BV test demonstrated a pooled sensitivity of 88% (95% CI: 81–93%) and a pooled specificity of 93% (95% CI: 91–94%) using the unstructured covariance model. No threshold effect was detected (ρ = −0.31).

**Figure 7:**
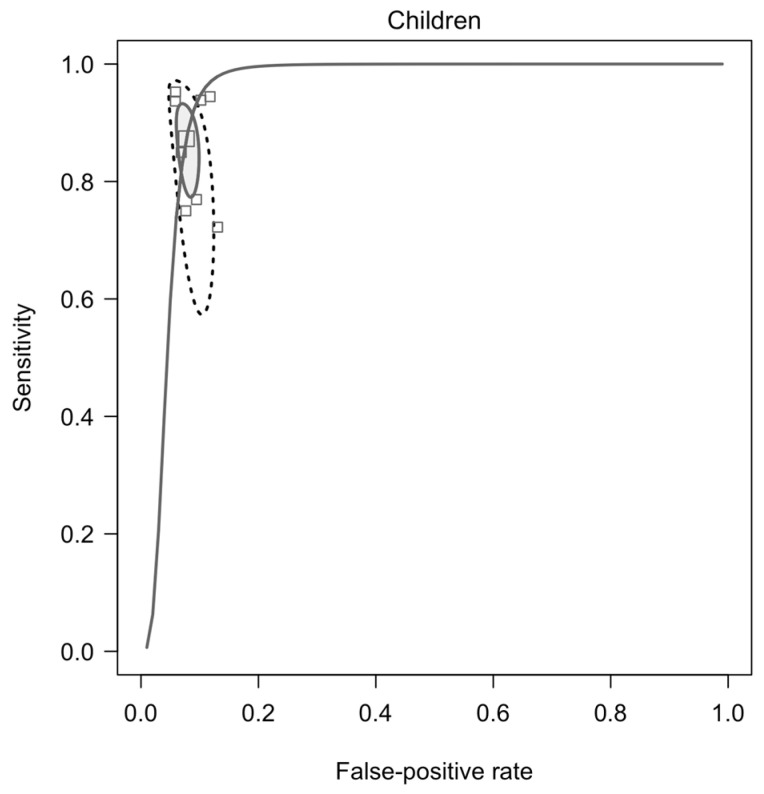
Pooled diagnostic accuracy of MeMed BV in children: HSROC model with unstructured covariance. The solid curve represents the fitted HSROC curve. The enclosed contours denote the confidence and prediction regions around the summary operating point. The gray background represents the default plotting area generated by Stata and has no statistical meaning.

The results from the HSROC model, performed as a supportive assessment of diagnostic performance, indicated a sensitivity of 69.9% and a specificity of 97.4% at a high-specificity operating point, yielding a DOR of 90.6 (95% CI: 47–173). The corresponding HSROC curve is shown in Figure 7. Importantly, this HSROC operating point reflects a threshold that prioritizes specificity and is not intended to represent the primary estimate of test performance. Given that all included studies used uniform manufacturer-defined thresholds, the bivariate model provides the most clinically relevant summary of performance. At these prespecified thresholds, diagnostic accuracy in children was comparable to that observed in adults, with overlapping confidence regions and no evidence of a meaningful difference between populations. Specificity remained very high, suggesting that false-positive results are uncommon in children, even in diagnostically challenging febrile presentations.

**Figure 8:**
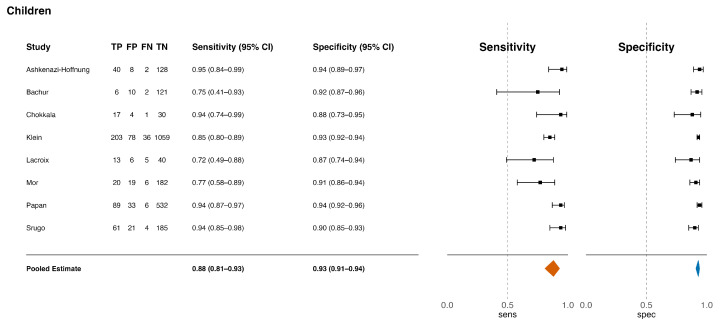
Coupled forest plot of study-level sensitivity and specificity estimates for the MeMed BV test (children). Black squares represent individual study estimates, and horizontal lines indicate 95% confidence intervals. The orange diamond denotes the pooled sensitivity estimate, and the blue diamond denotes the pooled specificity estimate.

Figure 8 presents a paired forest plot showing study-level estimates of sensitivity and specificity (with 95% CIs) for the MeMed BV test among pediatric participants across the included diagnostic accuracy studies. Sensitivity values ranged from 0.72 to 0.95 and specificity from 0.87 to 0.94, indicating high diagnostic performance with some variability across studies. Overall, the MeMed BV test demonstrated consistent accuracy in distinguishing bacterial from viral or non-bacterial infections among children presenting to emergency and urgent care settings, with sensitivity lower than that observed in the adult population.

### 3.5. Handling of Equivocal Results (Scenario E+)

There were several studies that chose to include participants whose MeMed BV test scores were within the equivocal range (35–65). A sensitivity analysis was conducted in which all equivocal results were treated as test-positive (Scenario E+). Under the bivariate random-effects model with unstructured covariance, the pooled estimates were

- Overall: sensitivity 92% (95% CI: 88–95%), specificity 82% (95% CI: 79–84%).
- Adults: sensitivity 94% (95% CI: 85–98%), specificity 82% (95% CI: 75–87%).
- Children: sensitivity 90% (95% CI: 85–94%), specificity 82% (95% CI: 80–85%).

Compared with the primary analysis, classifying equivocal results as positive yielded only a modest increase in pooled sensitivity, whereas specificity decreased substantially. Overall sensitivity increased from 91% to 92%, while specificity decreased from 92% to 82%. Similar patterns were observed across analyses of the adult and pediatric subgroups. These findings suggest that interpreting equivocal results as positive may slightly improve the detection of bacterial infections while increasing false-positive classifications.

### 3.6. Sensitivity Analyses: Risk of Bias (QUADAS-2)

To examine the influence of study risk of bias on diagnostic accuracy, a sensitivity analysis was conducted, including only the three pediatric studies assessed as having a low overall risk of bias (Allen et al. (2025) [28], Chokkalla et al. (2023) [31], Lacroix et al. (2023) [33]).

The pooled sensitivity was 84% (95% CI: 75–91%), and specificity was 86% (95% CI: 83–89%), with no measurable between-study heterogeneity (τ^2^ = 0.00; I^2^ = 0%). Sensitivities ranged from 72% [33] to 94% [31], and specificities ranged from 86% to 88% across all three datasets. The correlation coefficient (ρ = 0.97) indicated a high degree of consistency in study-level accuracy estimates.

These results suggest that the MeMed BV test maintained relatively high diagnostic performance even when restricting the analysis to methodologically robust pediatric studies, although pooled sensitivity and specificity estimates were somewhat lower than those observed in the overall pediatric meta-analysis (sensitivity 88% and specificity 91%). These findings indicate that methodological quality, including patient selection and handling of equivocal or indeterminate cases, may have influenced the magnitude of pooled diagnostic accuracy estimates.

### 3.7. Clinical Utility and Impact

Evidence from five non-DTA studies showed that MeMed BV can be used effectively in emergency departments and urgent care practices [19,36,37,38,39]. Table 6 summarizes key results from these five studies Overall, the five studies indicated that MeMed BV can influence clinical decision-making and be integrated into workflows without delaying care, with detailed findings for individual outcomes, including antibiotic prescribing, described below. Across settings, the test consistently influenced prescribing decisions by reducing antibiotic use when results suggested a viral infection and supporting timely treatment when results indicated a bacterial infection.

#### 3.7.1. Reduction in Unnecessary Antibiotic Use

Five studies assessed the impact of the MeMed BV test on antibiotic prescribing. Overall, these studies suggested that MeMed BV may influence prescribing behavior, particularly by reducing antibiotic use in patients with viral MeMed BV scores and increasing treatment in patients with bacterial scores [19,36,37,38,39]. However, the evidence is mainly based on process outcomes, and the pilot randomized trial by Singer et al. (2025), which was designed as a feasibility study and not powered for antibiotic prescribing endpoints, observed a non-statistically significant reduction in overall antibiotic prescribing [19].

In a pediatric emergency department, 79% of children with viral MeMed BV results were not prescribed antibiotics, aligning with physicians’ initial low prescribing intent [36]; notably, in that study, there was no evidence of adverse events associated with MeMed-guided decision-making in patients in whom antibiotics were withheld [36]. In a smaller urgent care pilot study, when physicians intended to prescribe antibiotics but MeMed BV indicated a viral infection, prescription rates dropped by 41%. In uncertain cases, clinicians followed MeMed BV guidance in almost 80% of patients [37].

In a large study among adult patients where physicians had planned to prescribe antibiotics, antibiotics were avoided in 63.1% cases with viral MeMed BV scores (397 of 629) [38]. Conversely, when the MeMed BV test identified bacterial infection in patients where no treatment was initially planned, antibiotics were started in 69.9% of the patients (283 of 405) [38]. The findings showed changes in antibiotic prescribing patterns, but it was unclear whether the changes reflected improved clinical decision-making or patient-centered clinical outcomes [38]. In the pilot randomized trial, overall antibiotic prescribing was lower in the MeMed BV group (24% vs. 30%, absolute difference of −6% (95% CI: −18% to 6%)) compared with standard care, which was statistically nonsignificant [19]. Patients with bacterial MeMed BV scores were more likely to receive antibiotics when the MeMed BV score was available (78% vs. 41%), while those with viral MeMed BV scores were less likely to be treated with antibiotics (12% vs. 25%) [19]. These findings suggest that the overall prescribing effect may reflect two opposing influences: reduced antibiotic use among patients with viral scores and increased prescribing among patients with bacterial scores, where antibiotics may otherwise have been withheld [19].

Additional observational evidence from pediatric patients suggested that MeMed BV results influenced antibiotic prescribing decisions. In this study, antibiotic prescribing aligned with MeMed BV results in 77.6% of cases overall and in 80.5% of non-referred patients, despite frequent pretest diagnostic uncertainty [39]. Viral MeMed BV results commonly led to withholding antibiotics even when clinicians were initially undecided or inclined to prescribe, while bacterial MeMed BV results prompted treatment when antibiotics had not been planned. Withholding antibiotics in patients with viral MeMed BV scores was not associated with increased 7-day hospitalizations or subsequent antibiotic prescriptions [39].

#### 3.7.2. Time to Result

Four studies reported results related to the time to result [19,37,38,39]. MeMed BV results were available during the acute care patient visit, indicating that they could be used in real-time. In both urgent care studies, more than 85% of clinicians reported that MeMed BV supported or changed their management decisions, indicating the ability to be integrated into routine workflows [37,38]. Kalmovich et al. (2026) reported that MeMed BV results were available within approximately one hour and concurrently with other point-of-care tests [39]. In the one pilot-randomized trial, four out of five patients were enrolled in emergency departments, where clinicians in the intervention arm received MeMed BV results to inform prescribing decisions [19].

#### 3.7.3. Hospitalizations

Data on hospitalizations were reported in a couple of studies, showing that having the MeMed BV test available also improved this longer-term outcome. In a large, observational, non-randomized decision-impact study conducted in urgent care centers, patients with bacterial MeMed BV scores who received antibiotics had fewer hospitalizations (7.8% vs. 30.3%) than patients with bacterial MeMed BV scores where antibiotics were withheld [38]. Further, in that same study, in approximately one in five cases, the test helped avoid unnecessary referrals to emergency departments [38]. Further, in the pilot randomized trial, patients randomized to the MeMed BV group had fewer return visits (3% vs. 8%) and no hospitalizations, compared with 3% in the control group [19].

#### 3.7.4. Other Outcomes

Included studies reported other outcomes related to clinicians’ impressions of the impact of the test on their decision-making and workflow integration. Physicians consistently reported that the test was useful in their decision-making, with one pilot study finding that 87% of clinicians stated that MeMed BV supported or changed their prescribing choices [37]. Similar impact was found in the larger follow-up study, with 86% of clinicians reporting that the test influenced and/or changed their antibiotic decision-making [38]. In another pediatric exploratory study, clinicians reported that MeMed BV supported clinical decision-making and considered the BV score to be a helpful tool in clinical practice, particularly when initial prescribing intent was uncertain [36]. Similarly, in a large study including pediatric patients, clinicians reported that MeMed BV supported or changed prescribing decisions in 82% of cases and discouraged emergency department referral in 26% of cases [39].

All five studies that described clinical impressions report that the MeMed BV test can be effectively integrated into everyday workflows in pediatric emergency departments or adult urgent care centers without disrupting care delivery [19,36,37,38,39].

### 3.8. Publication Bias

**Figure 9:**
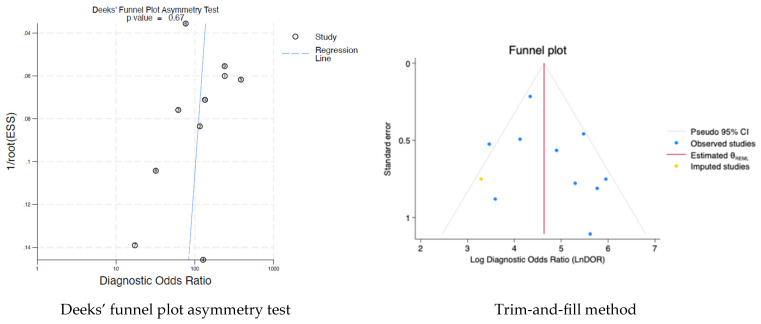
Funnel plots for publication bias (overall analysis). The (**left**) panel shows Deeks’ funnel plot asymmetry test, where the reported *p*-value (0.67) assesses funnel plot asymmetry; a *p*-value > 0.10 indicates no significant evidence of publication bias. The (**right**) panel shows the trim-and-fill analysis. Blue points represent observed studies, yellow points represent imputed studies, the red vertical line indicates the estimated pooled effect size (lnDOR), and the gray lines denote the pseudo 95% confidence limits.

Deeks’ funnel plot asymmetry test indicated no statistically significant association between study size and diagnostic accuracy (bias coefficient = −4.47, *p* = 0.67), suggesting no evidence of publication bias or small-study effects. Visual inspection of the funnel plot (Figure 9) showed mild asymmetry, with a slight clustering of smaller studies toward higher log diagnostic odds ratios (lnDOR). Using the trim-and-fill method, one potentially missing study was imputed on the left side of the plot, resulting in a minimal change in the pooled lnDOR from 4.74 (95% CI: 4.20–5.28) to 4.64 (95% CI: 4.10–5.17). These findings suggest that publication bias is unlikely to have affected the overall results, although interpretation is limited by the small number of included studies and the inherent challenges of publication bias assessment in diagnostic accuracy meta-analyses.

## 4. Discussion

### 4.1. Summary of Evidence

This systematic review and meta-analysis synthesized data from 16 studies evaluating the diagnostic accuracy and clinical utility of the MeMed BV test (and its manual predecessor, ImmunoXpert) for distinguishing bacterial from viral infections in acute-care settings. Across more than 6300 patients from Israel, Europe, and the United States, pooled estimates from 11 studies showed high and consistent diagnostic performance, with a sensitivity of 90% (95% CI: 86–93%) and a specificity of 91% (95% CI: 88–93%). These findings demonstrate reliable discrimination across heterogeneous populations and clinical contexts. Subgroup analyses confirmed comparable accuracy in adults (93%/89%) and children (88%/91%), with no threshold effect, indicating stable diagnostic behavior across age groups.

The earliest validation study by Oved et al. (2015) further supports these findings, which reported an AUC of 0.94 (95% CI: 0.92–0.96) with a sensitivity of 87% and specificity of 90% in a mixed-age cohort [12]. Although excluded from meta-analysis due to the use of different assay thresholds, its results reinforced the biological validity of the TRAIL/IP10/CRP host-response signature underlying MeMed BV. Evidence from four non-DTA studies complements these results: in one large prospective cohort (n = 3758) [38], antibiotics were prescribed in 20.6% of viral versus 73.2% of bacterial cases, while one pilot randomized trial showed lower prescribing rates (24% vs. 30%) and fewer return visits with BV-guided care [19]. Although the pilot randomized trial by Singer et al. (2025) reported lower overall antibiotic prescribing in the MeMed BV arm compared with standard care, the observed difference was not statistically significant [19]. However, this trial was designed as a feasibility study and was not powered to detect statistically significant differences in antibiotic prescribing [19]. Therefore, while the impact of MeMed BV on overall antibiotic prescribing and downstream antimicrobial stewardship outcomes remains uncertain, the absence of statistical significance should not be interpreted as a lack of effect.

These findings collectively demonstrate that MeMed BV delivers accurate, actionable information that improves diagnostic confidence and antibiotic stewardship. Most non-DTA studies included in this review primarily evaluated process-related outcomes, such as prescribing behavior and clinical decision-making, rather than direct patient-centered clinical outcomes. Accordingly, changes in prescribing patterns should not necessarily be interpreted as demonstrated improvements in broader clinical outcomes or antimicrobial stewardship effectiveness.

Although pooled analyses demonstrated relatively high diagnostic accuracy for MeMed BV, diagnostic accuracy alone does not establish incremental clinical utility beyond existing diagnostic approaches. Several included studies reported comparisons with CRP, procalcitonin, or routine laboratory parameters and generally found higher diagnostic performance with MeMed BV; however, comparative evidence across diverse clinical pathways and real-world diagnostic algorithms remains limited.

### 4.2. Comparison with Other Reviews and Diagnostic Biomarkers

The MeMed BV test consistently outperformed traditional inflammatory markers such as C-reactive protein (CRP) and procalcitonin (PCT). Direct comparisons reported AUCs greater than 0.90 for BV, markedly superior to 0.70–0.80 reported for PCT and CRP [17,20,28]. This enhanced accuracy likely reflects the integration of complementary immune pathways, including TRAIL and IP-10, which indicate antiviral activation, and CRP, which reflects the inflammatory burden, providing a more balanced view of the host response [12,33]. Such multi-analyte integration explains the stable performance of MeMed BV across age groups and infection syndromes, including undifferentiated fever and lower respiratory tract infections, where symptom overlap often complicates diagnosis.

The findings align with, but also extend, those of previous systematic reviews that have evaluated the performance and utility of point-of-care diagnostics for acute respiratory and febrile infections. The NIHR-commissioned overview of reviews by Webster et al. (2024) reported that most existing point-of-care tests and clinical prediction models demonstrate modest accuracy, with biomarker combinations achieving sensitivities of 80–90% and specificities of 82–93% [22]. However, the Webster et al. (2024) review did not include a quantitative synthesis of MeMed BV studies [22]. The current review thus represents the first meta-analysis of this specific host-response test, yielding narrower confidence intervals and demonstrating stability across adult and pediatric populations. Although HSROC analyses, included as supportive assessments, suggested a lower sensitivity at certain operating points in children, this reflects threshold prioritization rather than a consistent difference in performance, and primary analyses using manufacturer-defined thresholds demonstrated comparable accuracy across age groups.

These favorable findings contrast with prior meta-analyses of conventional biomarkers such as C-reactive protein (CRP) and procalcitonin (PCT), which showed only moderate and highly variable diagnostic accuracy in patients with lower RTI [40]. For CRP, sensitivity ranged from 52% to 90%, and specificity ranged from 42% to 91%, and for PCT, sensitivity ranged from 44% to 74%, and specificity ranged from 74% to 93%, highlighting poor reproducibility across thresholds and settings. In contrast, the pooled MeMed BV estimates (sensitivity: 90%, specificity: 91%) indicate higher and more consistent performance.

Carlton et al. (2021) assessed novel point-of-care biomarker combination tests [41]. For ImmunoXpert, the pooled sensitivity and specificity were 85% and 86% for bacterial infections, and 90% and 92% for viral infections, respectively [41]. These results are consistent with, but less precise than, the present review, which incorporates the most up-to-date evidence and a larger dataset, which included automated MeMed BV studies.

While MeMed BV demonstrates clear analytical and operational advantages, the evidence must be interpreted in light of several methodological considerations.

### 4.3. Strengths and Limitations

This review has notable strengths. It provides the most comprehensive synthesis to date of diagnostic accuracy and clinical utility data for MeMed BV, including studies conducted over nearly a decade across multiple regions. The review followed a predefined protocol and applied a rigorous search strategy across MEDLINE, Embase, and Cochrane Library, with standardized data extraction and bias assessment (QUADAS-2, Cochrane RoB 2.0, and JBI checklists). Duplicate screening and independent verification minimized reviewer bias, while bivariate random-effects modeling appropriately accounted for correlation between sensitivity and specificity.

The review employed rigorous, pre-specified methods and comprehensive search strategies to ensure that all relevant evidence was captured. This approach represents a major strength, providing transparency and minimizing the risk of overlooking important studies. The risk of bias in the primary studies was systematically assessed using established, validated tools appropriate for each study design. Additionally, potential sources of heterogeneity were evaluated across populations, settings, and reference standards.

Another strength lay in the identification and description of limitations in the underlying evidence base, including concerns related to study design and reporting. The use of advanced statistical approaches, including bivariate and HSROC models, with structured risk-of-bias assessments, enhanced the robustness of the analysis. By integrating comprehensive evidence retrieval with systematic appraisal of study quality and heterogeneity, the review provides a rigorous and reliable synthesis currently available on the diagnostic performance of the MeMed BV test.

In terms of the limitations, at the study level, most DTA studies were prospective and cross-sectional, utilizing manufacturer thresholds and blinded expert adjudication [28,29,31], which supports internal validity. However, it is important to note that the flow and timing domain was often rated high-risk due to the exclusion of equivocal MeMed BV results (35–65), which may inflate accuracy. The flow and timing domain assesses whether all enrolled patients were included in the analysis and whether any exclusions or delays could bias results. However, sensitivity analysis treating these as positive (Scenario E+) corroborates the trade-off, with a sensitivity of 92% and a specificity of 81%. In addition, reference standards varied, combining microbiological confirmation and expert adjudication, which mirrors clinical reality but introduces the potential for misclassification [20,33]. Several studies employed convenience sampling or mixed inpatient populations, which limits generalizability to frontline urgent-care contexts.

An additional methodological consideration relates to the use of expert adjudication as the reference standard in most included studies. Although this approach was commonly used in studies evaluating bacterial versus viral infection because no single gold standard exists, adjudication decisions frequently incorporated clinical findings, microbiological testing, inflammatory biomarkers, imaging results, and follow-up information.

Reference-standard heterogeneity should be considered when interpreting the pooled estimates. As illustrated by the analysis of Allen et al. (2025), which explored multiple adjudication-based reference standard definitions with varying levels of diagnostic certainty, estimates of diagnostic performance may vary depending on how uncertainty is classified [28]. Although this study was not included in the meta-analysis because it represents a secondary pooled analysis rather than an independent primary diagnostic accuracy study, its findings were directionally consistent with the studies included in this review, despite differences in reference standard definition. This alignment suggests that variability in diagnostic accuracy estimates across studies is more likely attributable to differences in how diagnostic uncertainty is handled rather than to intrinsic differences in test performance. The included studies used microbiological confirmation, expert adjudication, or hybrid definitions to classify infection etiology. Although these approaches are clinically reasonable given the absence of a universally accepted gold standard for differentiating bacterial from viral infection [13,14], they may classify some cases differently, which could have influenced the pooled sensitivity and specificity estimates.

An additional limitation was that pooled analyses were stratified by age group rather than clinical syndrome. Although the included studies commonly enrolled patients with RTIs, UTIs, or undifferentiated fever, several studies included mixed infectious presentations and did not consistently report extractable syndrome-specific diagnostic accuracy estimates. Therefore, syndrome-specific meta-analyses were not feasible, and the generalizability of pooled estimates across distinct clinical syndromes should be interpreted cautiously.

Sensitivity analyses restricted to studies at lower risk of bias yielded slightly lower pooled diagnostic accuracy estimates, particularly in pediatric populations. These findings suggest that methodological quality, including patient selection and handling of equivocal or indeterminate cases, may have influenced the magnitude of pooled estimates. However, the MeMed BV test continued to demonstrate relatively high diagnostic performance across analyses.

Several included studies disclosed industry funding or manufacturer involvement. Although the funding source is not included as a formal domain within the QUADAS-2 framework [23], it remains an important contextual consideration when interpreting the overall strength of evidence. While evidence regarding sponsorship bias in diagnostic accuracy studies remains limited, findings from broader evaluations of industry-sponsored drug and device research suggest that the funding source should be considered when interpreting the evidence base [42].

Assessment of publication bias in DTA meta-analyses remains methodologically challenging. While Deeks’ test was used as the primary formal assessment, all publication bias results should be interpreted with caution, given the inherent limitations of asymmetry-based methods in the DTA setting.

At the review level, comprehensive searches and duplicate processes help minimize bias; however, the incomplete retrieval of unpublished or non-English data remains a possibility. A small number of large multicenter trials constrained formal assessment of publication bias. Moreover, this review focused primarily on diagnostic accuracy and behavioral outcomes; economic and patient-centered endpoints were out of scope and warrant further evaluation.

### 4.4. Implications for Clinical Practice and Future Research

The accumulated evidence supports MeMed BV as a rapid, evidence-based adjunct to clinical judgment for guiding antibiotic decisions in acute-care settings. Providing results within 15 min, it enables same-encounter differentiation between bacterial and viral infections, a critical clinical conundrum faced daily by acute care physicians, and potentially reduces unnecessary antibiotic exposure and supports antimicrobial stewardship goals [19,38]. The integration of BV testing into emergency workflows could optimize patient management and resource utilization without compromising safety.

Equivocal MeMed BV results should be interpreted alongside the overall clinical assessment and other available diagnostic information. Several included studies noted that equivocal results may occur in situations with overlapping host-response patterns, evolving infection, bacterial–viral co-infection, or uncertainty within the reference standard itself [12,17,31]. In these situations, repeating the clinical assessment and integrating it with routine laboratory, microbiological, and radiological findings remains important for clinical decision-making.

An additional consideration is that MeMed BV was developed to support differentiation between bacterial and viral/non-bacterial infectious etiologies and has not been specifically validated as a diagnostic tool for non-infectious inflammatory disorders. Several included studies highlighted the complexity of host response patterns and the potential for overlap between infectious and inflammatory states in clinically heterogeneous patient populations [12,17]. Accordingly, MeMed BV results should be interpreted alongside clinical evaluation, patient history, and other laboratory, microbiological, and radiological investigations.

Future research should extend to populations with altered immune responses, such as immunocompromised, elderly, or multimorbid patients, and evaluate the long-term impact of MeMed BV-guided care on patient outcomes, antibiotic resistance, and healthcare costs. Methodological standardization, particularly regarding treatment of equivocal MeMed BV scores and use of composite reference standards, will improve comparability. Comparative studies with emerging molecular or host-response platforms, when reported in peer-reviewed publications, will further clarify the test’s role within evolving diagnostic algorithms for acute infections.

## 5. Conclusions

This systematic review demonstrates that the MeMed BV test accurately distinguishes between bacterial and viral infections in adults and children. Diagnostic performance was consistent across diverse study designs and clinical settings and was supported by evidence of reductions in unnecessary antibiotic prescribing, with favorable trends and no reported adverse events. MeMed BV represents a validated, rapid, and implementable diagnostic tool with substantial potential to improve clinical decision-making and strengthen antimicrobial stewardship efforts in emergency and urgent care settings.

## Figures and Tables

**Table 1:** Characteristics of included DTA studies.

Study ID	Study Design	Population	Sample Size	Index Test	Reference Standard	Funding Source
Allen 2025 [28]	Multi-cohort diagnostic accuracy study	Patients older than 90 days with suspected acute bacterial or viral infection presenting to the ED/UCC. Adults: 57.5% Children: 42.5% Median age (IQR): 25 years (4–47) Percentage of females: 49.9%	Total enrolled: 1016 ^a^ Actionable MeMed BV results (%): 914/1016 (89.96%) Equivocal MeMed BV results (%): 102/1016 (10.04%)	MeMed BV^®^ test performed on the MeMed Key^®^ and ImmunoXpert™ platforms	Clinical adjudication was conducted in 3 rounds by 21 international clinicians	The study population was drawn from 3 prospective studies. The Autopilot study was supported by the H2020 Fast Track to Innovation, Observer study was supported by the H2020 Innovation in SMEs. Apollo study: MeMed provided funding to the participating clinical sites.
Ashkenazi-Hoffnung 2018 [29]	Cross-sectional diagnostic accuracy study	Pediatric and adult patients presenting with respiratory infection (upper or lower) or fever without source, presenting to the ED or hospital (majority ED) Adults: 35% Children: 65% Mean age of children (SD): 4.1 years (4.0) Mean age of adults (SD): 49.8 years (19.5) Percentage of females: 42%	Total enrolled: 314 ^b^ Actionable MeMed BV results (%): 282/314 (89.8%) Equivocal MeMed BV results (%): 32/314 (10.2%)	MeMed BV^®^ test performed on ImmunoXpert™ platform	Expert panel adjudication in line with NHS Health Technology Assessment guidelines (Three independent, experienced, clinically practicing physicians)	Industry-funded—the work was supported by funds from MeMed, which financed the index tests.
Bachur 2024 [20]	Cross-sectional diagnostic accuracy study	Patients aged over 90 days with a clinician’s suspicion of an acute bacterial or viral infection presenting to the ED/UCC Adults: 66% Children: 34% Mean age (SD): 27.9 years (19.4) Percentage of females: 52.3%	Total enrolled: 476 Indeterminate results: 60/476 Actionable MeMed BV results (%): 386/416 (92.8%) Equivocal MeMed BV results (%): 30/416 (7.2%)	MeMed BV^®^ test performed on MeMed Key^®^ platform	Expert adjudication by three experienced, clinically practicing physicians	Industry-funded—the study was funded by MeMed.
Cabañas Morafraile 2024 [30]	Cross-sectional diagnostic accuracy study	Patients aged ≥ 18 years, clinically diagnosed with an acute infection in the ED. Mean age (SD): 68.28 years (19.53) Percentage of females: 42.6%	Total enrolled: 258 Indeterminate results: 17/258 Actionable MeMed BV results (%): 217/241 (90.1%) Equivocal MeMed BV results (%): 24/241 (9.9%)	MeMed BV^®^ test performed on the LIAISON^®^ platform	Microbiological tests	The reagents for the LIAISON MeMed BV test were provided by DiaSorin. No financial funding was received for the study. The company had no role in study design, data analysis, interpretation, or manuscript preparation.
Chokkalla 2023 [31]	Cross-sectional diagnostic accuracy study	Pediatric patients aged ≤ 18 with acute febrile illness visited the ED and had a CRP test requested. Mean age (SD): 6.9 (9.25) Percentage of females: 58%	Total enrolled: 60 ^a^ Actionable MeMed BV results (%): 52/60 (86.7%) Equivocal MeMed BV results (%): 8/60 (13.3%)	MeMed BV^®^ test performed on the MeMed Key^®^ platform	Microbial confirmation by PCR or blood cultures, and clinical adjudication	NR
Halabi 2023 [17]	Cross-sectional diagnostic accuracy study	Patients aged 18 years or older presenting to the ED with clinical suspicion of LRTI Median age (IQR): 56 years (35) Percentage of females: 45.3%	Total enrolled: 415 Indeterminate results: 101/415 Actionable MMBV results (%): 284/314 (90.4%) Equivocal MMBV results (%): 30/314 (9.6%)	MeMed BV^®^ test performed on the ImmunoXpert™ platform	Expert panel adjudication (at least two experts)	This work was supported by the European Commission under H2020-SMEINST-2-2015 (grant 684589).
Klein 2023 [32]	Cross-sectional diagnostic accuracy study	Pediatric patients aged 3 months to 18 years with symptoms of acute infection at the ED or pediatric ward (majority ED) Median age (IQR): 1.8 years (3.7) Percentage of females: 46%	Total enrolled: 2155 Indeterminate results: 594/2155 Actionable MeMed BV results (%): 1376/1561 (88.1%) Equivocal MeMed BV results (%): 185/1561 (11.9%)	The MeMed BV^®^ test performed on the ImmunoXpert™ platform	Expert Adjudication in accordance with the National Health Service (NHS) Health Technology Assessment Guidelines for Evaluation of Diagnostic Tests (3 pediatricians)	No external funding received.
Lacroix 2023 [33]	Cross-sectional diagnostic accuracy study	Children with undifferentiated fever aged less than 3 years old presenting to the PED Median age (IQR): 2 months (1–4.9) Percentage of females: 39%	Total enrolled: 241 ^c^ Actionable MeMed BV results (%): 64/75 (85.3%) Equivocal MeMed BV results (%): 11/75 (14.7%)	MeMed BV^®^ test performed on the ImmunoXpert™ platform	Expert panel adjudication (one senior pediatric infectious-disease specialist and two senior pediatric emergency physicians	Investigator-driven study. Alain Gervaix received financial support from MeMed.
Mor 2023 [34]	Cross-sectional diagnostic accuracy study	Children aged 3 months to 18 years old with clinical suspicion of RTI, undifferentiated fever, UTI, or gastroenteritis presenting to PED. Median age (IQR): 1.3 years (1.7) Percentage of females: 39.7%	Total enrolled: 287 Indeterminate results: 34/287 Actionable MeMed BV results (%): 227/253 (89.7%) Equivocal MeMed BV results (%): 26/253 (10.3%)	MeMed BV^®^ test performed on ImmunoXpert™ platform	Expert adjudication in line with the National Health Service (NHS) Health Technology Assessment Guidelines for Evaluation of Diagnostic Tests (Three pediatricians with more than 7 years of experience)	MeMed funded the study for laboratory assistants, study assistants, and shipping.
Oved 2015 [12]	Two-group Diagnostic accuracy study	Pediatric patients (18 years) and adults (>18 years) with clinical suspicion of an acute infectious disease presenting to the ED, pediatric wards, and internal medicine departments (majority ED) Percentage of females: 47% Adults: 44% Children: 56%	Total enrolled: 765 ^b^ Controls: 112/765 Actionable MeMed BV results (%): 567/653 (86.8%) Equivocal MeMed BV results (%): 86/653 (13.1%)	MeMed BV^®^ test performed on ImmunoXpert™ platform	Expert panel adjudication	This study was funded by MeMed.
Papan 2022 [35]	Cross-sectional diagnostic accuracy study	Children aged between 90 days and 18 years, presenting to PED with clinical suspicion of RTI or undifferentiated fever. Mean age (SD): 3.5 years (3.6) Percentage of females: 43.1%	Total enrolled: 1008 Indeterminate results: 276/1008 Actionable MeMed BV results (%): 660/732 (90.2%) Equivocal MeMed BV results (%): 72/732 (9.8%)	MeMed BV^®^ test performed ImmunoXpert™ platform	Expert panel adjudication (Pediatricians with >10 years of clinical experience)	This study was supported by the European Commission, Executive Agency for Small and Medium-sized Enterprises, Horizon2020 FTIPilot-2015-1 program (grant number 701088).
Srugo 2017 [13]	Cross-sectional diagnostic accuracy study	Pediatric patients with clinical and radiological pneumonia, or fever without an identified source, presenting to the pediatric ED and pediatric wards (majority ED) Mean age (SD): 4.1 years (4.2) Percentage of females: 47%	Total enrolled: 361 Indeterminate results: 54/361 Actionable MeMed BV results (%): 271/307 (88.3%) Equivocal MeMed BV results (%): 36/307 (11.7%)	MeMed BV^®^ test performed ImmunoXpert™ platform	Expert panel adjudication	This was an investigator-driven study. MeMed funded and conducted the ImmunoXpert™ assays.

Abbreviations: AUC, area under curve; BM, biomarker; CRP, C-reactive protein; DOR, diagnostic odds ratio; ED, emergency department; LR+, positive likelihood ratio; LR−, negative likelihood ratio; LRTI, lower respiratory tract infection; MMBV, MeMed BV test; NPV, negative predictive value; PCT, procalcitonin; PED, pediatric emergency department; PPV, positive predictive value; RTI, respiratory tract infection; UCC, urgent care center; UTI, urinary tract infection; URTI, upper respiratory tract infection. The reported sample sizes for the total enrolled patients reflect both indeterminate reference standard cases and equivocal index test results, and the characteristics of patients (% females, median age) are based on these. Actionable MeMed BV results refer to index test results classified as either positive or negative (a term used in some of the included studies). Patients with equivocal MeMed BV results or an indeterminate reference standard were excluded from the meta-analysis of sensitivity and specificity, consistent with the populations analyzed in the included studies. ^a^: The adjudication process did not define a separate indeterminate category for the reference standard. ^b^: Indeterminate results were excluded prior to patients’ enrollment. Ashkenazi-Hoffnung 2018 [29] and Oved 2015 [12] excluded 116 and 98 results, respectively. ^c^: Data presented for a mixed population including patients aged less than 90 days.

**Table 2:** Characteristics of included non-DTA studies.

Study ID	Study Design	Population	Sample Size	Index Test	Reference Standard	Funding Source
Fröhlich 2023 [36]	Prospective cohort study	Children and adolescents aged > 90 days with respiratory tract infection symptoms or fever without an apparent focus at the pediatric ED Median age (IQR): 3.1 years (1.3–4.3) Percentage of females: 54.7%	Study cohort: 53 Actionable MeMed BV results (%): 42/53 (79.2%) Equivocal MeMed BV results (%): 11/53 (20.8%)	MeMed BV^®^ test performed on MeMed Key^®^ platform	Clinical adjudication (Three pediatricians)	The study was funded by a 2021 CAREer Grant from the European Society of Clinical Microbiology and Infectious Diseases (ESCMID). Additionally, it was supported by a grant awarded to MeMed from the European Commission, Executive Agency for Small and Medium-sized Enterprises H2020-EIC-SMEInst-2018-2020-2 [grant number 88124]
Kalmovich 2023 [37]	Prospective non-comparative study	Adult and pediatric patients presenting to UCCs with suspected acute bacterial or viral infection Adults: 42% Children: 58% Median age (IQR): 6 years (1.5–38.3) Percentage of females: 51%	Study cohort: 152 Actionable MeMed BV results: 131/152 (86.2%) Equivocal MeMed BV results (%): 21/152 (13.8%)	MeMed BV^®^ test performed on MeMed Key^®^ platform	NA	This research received no external funding
Kalmovich 2025 [38]	Prospective non-comparative study	Adult patients presenting to UCCs with fever and upper RTI Median age (IQR): 42 years (31–58) Percentage of females: 59%	Study cohort: 3758 Actionable MeMed BV results (%): 3262/3758 (86.8%) Equivocal MeMed BV results (%): 496/3758 (13.2%)	MeMed BV^®^ tests performed on the MeMed Key^®^ platform	NA	The study was conducted without external funding
Kalmovich 2026 [39]	Prospective non-comparative study	Pediatric patients presenting to UCCs with suspected acute infection Median age (IQR): 3 years (2–6) Percentage of females: 51.4%	Study cohort: 2016 Actionable MeMed BV results (%): 1784/2016 (88.5%) Equivocal MeMed BV results (%): 232/2016 (11.5%)	MeMed BV^®^ test performed on MeMed Key^®^ platform	NA	Not reported
Singer 2025 [19]	Randomized controlled trial	Adult (≥18 years) patients presenting at ED/UCC with symptoms of LRTI Median age (IQR): 40 years (28–55.8) Percentage of females: 57%	Study cohort: 214 Actionable MMBV results (%): 184/212 (86.8%) Equivocal MeMed BV results (%): 28/212 (13.2%) SC arm: 106 MeMed BV arm: 108	MeMed BV^®^ test performed on MeMed Key^®^ platform	NA	This study was partially supported by federal funds from the Department of Health and Human Services, Administration for Strategic Preparedness and Response, Biomedical Advanced Research and Development Authority (BARDA), under contract number 75A50123C00041. Additionally, MeMed was a primary sponsor of this study, and DiaSorin Inc. was a co-sponsor.

Abbreviations: CI, confidence interval; ED, emergency department; IQR, interquartile range; MMBV, MeMed BV test; NA, not applicable; UCC, urgent care center. Actionable MeMed BV results refer to index test results classified as either positive or negative (a term used in some of the included studies).

**Table 3:** Risk of bias summary table.

Study	Tool	Overall RoB	Summary
Singer 2025 [19]	Cochrane RoB 2.0	Low	Robust randomization, blinding; minimal concerns
Kalmovich 2023 [37]	ROBINS-I	Serious	No independent control group; clinician-directed test ordering and lack of adjustment introduced confounding; reliance on clinician-reported intention and perceived test impact contributed to measurement bias
Frohlich 2023 [36]	JBI cohort	Moderate	Confounding, unclear follow-up
Kalmovich 2025 [38]	ROBINS-I	Serious	Lacks a comparator group; discretionary test ordering introduced confounding by indication, and reliance on clinician-reported outcomes and incomplete questionnaire data increased the risk of measurement and missing-data bias
Kalmovich 2026 [39]	ROBINS-I	Serious	Non-comparative study; discretionary clinician-directed test ordering introduced confounding by indication and selection bias; clinician-reported influence of test results introduced subjective measurement bias

**Table 4:** Pooled diagnostic performance metrics.

Population	Number of Studies	Sensitivity (%), (95% CI)	Specificity (%), (95% CI)	Between-Study Variance (Tau^2^)
Adults	4	93 (87–97)	91 (88–94)	0.00
Children	8	88 (81–93)	93 (91–94)	0.00
Overall (Adults + children)	10	91 (86–94)	92 (91–93)	0.01

**Table 5:** Overall diagnostic accuracy of MeMed BV in individual studies.

Study (Year)	Total Number of Patients Analyzed	Sensitivity (%), (95% CI)	Specificity (%), 95% CI	AUC (95% CI)
Ashkenazi-Hoffnung 2018 [29]	282	93 (88–97)	94 (90–97)	Not reported
Bachur 2024 [20]	386	90 (77–96)	93 (90–95)	0.95 (0.90–0.99)
Cabañas Morafraile 2024 [30]	217	87 (81–92)	90 (80–95)	0.92 (0.88–0.96)
Chokkalla 2023 [31]	52	94 (74–99)	88 (73–95)	Not reported
Halabi 2023 [17]	284	98 (93–99)	88 (83–92)	Not reported
Klein 2023 [32]	1376	85 (80–89)	93 (92–94)	Not reported
Lacroix 2023 [33]	64	72 (49–88)	87 (74–94)	0.86 (0.76–0.96)
Mor 2023 [34]	227	77 (58–89)	91 (86–94)	0.91 (0.84–0.99)
Papan 2022 [35]	660	94 (87–97)	94 (92–96)	Not reported
Srugo 2017 [13]	271	94 (85–98)	90 (85–93)	0.96 (0.92–0.99)

**Table 6:** Key results from non-DTA studies.

Study ID	Population	Key Results
Fröhlich 2023 [36]	Children and adolescents aged over 90 days with symptoms of respiratory tract infection or fever without an apparent focus. Sample size: Total enrolled: 111, Total analyzed: 53 Median age (IQR): 3.1 years (1.3–4.3) Percentage of females (n): 54.7% (29)	The median MeMed BV score in the viral (non-COVID-19) group was 9 (IQR, 0–25.5); the bacterial group had a median score of 66 (IQR, 22–81.5). Experts aligned with MeMed BV results in 9/15 cases (64.3%). Physicians considered the MeMed BV score to be a helpful tool in clinical practice for confirming treatment decisions (n = 22, 52%) or changing therapy (12%).
Kalmovich 2023 [37]	Adult and pediatric patients presenting to UCCs with a suspected acute bacterial or viral infection. Sample size: Total enrolled: 152, Total analyzed: 131 Adults: 64 Children: 88 Median age (IQR): 6 years (1.5–38.3) Percentage of females (n): 51% (77)	Physician prescribing patterns showed that MeMed BV testing reduced unnecessary antibiotic use. In cases of diagnostic uncertainty, doctors followed MeMed BV results in 78.9% of patients, treating bacterial cases and avoiding antibiotics for most viral cases. The MeMed BV score had a positive impact on the decision-making process.
Kalmovich 2025 [38]	Adult patients presenting to UCCs with fever and upper RTI Sample size: Total enrolled: 3758, Total analyzed: 3262 Median age (IQR): 42 years (31–58) Percentage of females (n): 59% (2228)	Antibiotics were prescribed in 1364/3758 (36.3%) of cases, with a higher prescription rate for bacterial cases (628/858, 73.2%) than for equivocal (241/496, 48.6%) and viral (495/2404, 20.6%) cases. ED referral rates were 19.3% for bacterial cases, 11.1% for equivocal cases, and 7.2% for viral cases. Physicians adhered to MeMed BV results regarding antibiotic prescriptions in 77.8% (2537/3262) of cases.
Kalmovich 2026 [39]	Pediatric patients presenting to UCCs with fever and upper and lower RTIs Sample size: Total enrolled: 2171, Total analyzed: 2016 Median age (IQR): 3 years (2–6) Percentage of females (n): 51.4% (1037)	MeMed BV results influenced or supported antibiotic prescribing decisions in 82.0% of cases and aligned with prescribing decisions in 77.6% overall (80.5% among non-referred patients). MeMed BV results discouraged ED referral in 26.0% of cases without increasing 7-day hospitalization rates. Antibiotic prescribing was reduced in viral MeMed BV cases and increased in bacterial MeMed BV cases, with no significant differences in post-visit hospitalizations or antibiotic use during follow-up, supporting the safe clinical utility of MeMed BV in pediatric urgent care settings.
Singer 2025 [19]	Adult (≥18 years) patients presenting at ED/UCC with symptoms of LRTI. Sample size: Total enrolled: 260, Total analyzed: 212 Median age (IQR): 40 years (28–55.8) Percentage of females (n): 57% (122)	The antibiotic prescription rate was 30% (95% CI: 22% to 40%) in the SC arm and 24% (95% CI: 17% to 33%) in the MeMed BV arm. This represents an absolute difference of −6% (95% CI: −18% to 6%) and a relative difference of −20% (95% CI: −49% to 24%).

## Data Availability

No new datasets were generated or analyzed in this study. All data were obtained from previously published studies cited in the manuscript.

## Supplementary Materials

The following supporting information can be downloaded at: https://www.mdpi.com/article/10.3390/diagnostics16121930/s1.

## Abbreviations

The following abbreviations are used in this manuscript:

ANC	Absolute neutrophil count
AUC	Area under the curve
CDC	Centers for Disease Control and Prevention
CI	Confidence interval
CRP	C-reactive protein
DTA	Diagnostic test accuracy
DOR	Diagnostic odds ratio
ED	Emergency department
IP-10	IP-10 interferon gamma-induced protein 10
HSROC	Hierarchical summary receiver operating characteristic
IQR	Interquartile range
LRTI	Lower respiratory tract infection
NPV	Negative predictive value
PCT	Procalcitonin
PPV	Positive predictive value
PRISMA-DTA	Preferred Reporting Items for Systematic Reviews and Meta-Analyses of Diagnostic Test Accuracy
PROSPERO	International Prospective Register of Systematic Reviews
QUADAS-2	Quality Assessment of Diagnostic Accuracy Studies-2
REML	Restricted maximum likelihood
RoB 2.0	Risk of Bias 2 tool
ROBINS-I	Risk of Bias in Non-randomized Studies of Interventions
ROC	Receiver operating characteristic
RTI	Respiratory tract infection
TRAIL	Tumor necrosis factor-related apoptosis-inducing ligand
UCC	Urgent care center
URTI	Upper respiratory tract infection
UTI	Urinary tract infection
WHO	World Health Organization

## Appendix A. Full Search Strategies in All Four Databases

**Database**	**Search Strategy**	**Number of Hits**
Medline and Medline In-Process	“MeMed”[tiab] OR “MMBV”[tiab] OR “ImmunoXpert”[tiab] OR “BV score”[tiab] OR “TRAIL, IP-10, CRP”[tiab] OR “host-response proteins”[tiab] OR “host-response biomarkers”[tiab] OR “TRAIL/IP-10/CRP”[tiab] OR “host biomarkers”[tiab] OR “immune-protein score”[tiab] OR “multi-protein signature”[tiab] OR “host protein signature”[tiab] OR “host signature”[tiab] OR “host test”[tiab] OR “immune-protein signature”[tiab] OR “combined biomarker signature”[tiab]	414
Embase	(MeMed OR MMBV OR ImmunoXpert OR “BV score” OR “TRAIL, IP-10, CRP” OR “host-response proteins” OR “host-response biomarkers” OR “TRAIL IP-10 CRP” OR “host biomarkers” OR “immune-protein score” OR “multi-protein signature” OR “host protein signature” OR “host signature” OR “host test” OR “immune-protein signature” OR “combined biomarker signature”).ti,ab.	580
CINAHL	TI (MeMed OR MMBV OR ImmunoXpert OR “BV score” OR “TRAIL, IP-10, CRP” OR “host-response proteins” OR “host-response biomarkers” OR “TRAIL/IP-10/CRP” OR “host biomarkers” OR “immune-protein score” OR “multi-protein signature” OR “host protein signature” OR “host signature” OR “host test” OR “immune-protein signature” OR “combined biomarker signature”) OR AB (MeMed OR MMBV OR ImmunoXpert OR “BV score” OR “TRAIL, IP-10, CRP” OR “host-response proteins” OR “host-response biomarkers” OR “TRAIL/IP-10/CRP” OR “host biomarkers” OR “immune-protein score” OR “multi-protein signature” OR “host protein signature” OR “host signature” OR “host test” OR “immune-protein signature” OR “combined biomarker signature”)	63
Cochrane Library	MeMed:ti,ab OR MMBV:ti,ab OR ImmunoXpert:ti,ab OR “BV score”:ti,ab OR “TRAIL, IP-10, CRP”:ti,ab OR “host-response proteins”:ti,ab OR “host-response biomarkers”:ti,ab OR TRAIL/IP-10/CRP:ti,ab OR “host biomarkers”:ti,ab OR “immune-protein score”:ti,ab OR “ multi-protein signature”:ti,ab OR “host protein signature”:ti,ab OR “host signature”:ti,ab OR “host test”:ti,ab OR “immune-protein signature”:ti,ab OR “combined biomarker signature”:ti,ab	28

## Appendix B. List of Excluded Studies with Reasons for Exclusion

**First Author**	**Publication Year**	**PubMed ID** **(PMID)**	**Reason for Exclusion**
Ashkenazi-Hoffnung	2021	34966702	Patients were recruited from hospitalized inpatients, with no evidence of involvement in an ED or urgent care setting. It does not meet the setting criteria.
Dedeoglu	2024	39581271	The study was conducted entirely among hospital inpatients. All participants were adults hospitalized with ARI, recruited only after admission. The study did not assess diagnostic accuracy or clinical utility in ED or urgent care settings among patients with suspected RTI or fever of unknown origin.
Denina	2025	39535736	The publication type is a Letter to the Editor, describing a small, single-center observational experience that is explicitly excluded by the protocol’s criteria. It provides minimal details on study results. In addition, the study population is limited to children with community-acquired pneumonia, including ~20% mycoplasma pneumoniae cases, with no separate diagnostic accuracy results reported specifically for RTI or fever of unknown origin, excluding mycoplasma infections.
Hainrichson	2023	35487256	This is an analytical validation study focused on laboratory precision, sensitivity, linearity, stability, and platform comparison of the MeMed BV test. No recruitment of patients with RTI or fever of unknown origin presenting to ED/UCC settings. It does not report diagnostic accuracy or clinical utility outcomes against a reference standard in the target population.
Langedijk	2024	38306791	The study involved healthy adult volunteers with no recent or current infection. It assessed biomarker variability in the uninfected population without evaluating diagnostic accuracy or clinical utility in patients with RTI or fever of unknown origin.
Noni	2025	40150663	The study was conducted entirely in hospitalized pediatric inpatients (a 100% inpatient population), which does not meet the inclusion criteria, as it is limited to emergency department or urgent care settings.
Novak	2023	36437179	A retrospective descriptive chart review and clinician survey study design without a formal cohort structure or diagnostic accuracy evaluation; it does not meet the specified inclusion criteria for study designs.
Papan	2023	36043485	The study was conducted predominantly among hospitalized pediatric inpatients (~73%) and did not report primary recruitment from emergency department or urgent care settings, thereby not meeting the protocol inclusion criteria.
Papan	2024	38092364	The study included more than 50% hospitalized inpatients (53.8%) and did not demonstrate primary recruitment from emergency or urgent care settings, which does not meet the protocol eligibility criteria.
Portefaix	2022	36362791	This study included children with clinically suspected serious bacterial infection, excluding general undifferentiated fever or RTI presentations. It did not evaluate the MeMed BV test (TRAIL, IP-10, CRP signature) but instead developed new models using multiple biomarkers (Used TRAIL, IP-10, CRP as inputs along with four other biomarkers (PCT, IL-6, NGAL, MxA)).
Ramgopal	2024	39397543	This study was a secondary analysis of another study, rather than an original primary study.
Shapira	2018	30091387	This laboratory methods study described an automation of the ELISA assay protocol without patient recruitment, clinical diagnostic accuracy outcomes, or clinical utility assessment in RTI or febrile populations. Patients with RTI or fever of unknown origin were not recruited in a clinical care setting. Only de-identified stored serum samples were used for assay verification.
Steckiewicz	2024	38490312	The study enrolled ICU inpatients (100%) exclusively, meeting the protocol exclusion criterion for studies conducted predominantly in intensive care unit settings. There were mixed causes for ICU admission, with no separate outcomes for RTI or fever of unknown origin, and no diagnostic accuracy or clinical utility data from ED or urgent care settings.
Stein	2018	29273482	The study recruited both ED and hospital ward patients with suspected LRTI but did not report the proportion of inpatients. Unable to confirm that patient recruitment from the ED exceeded the 50% threshold specified in the eligibility criteria.
Stein	2022	36389361	The study is a sub-analysis limited to pediatric patients with PCR-confirmed adenovirus infection, excluding other viral and bacterial causes of RTI or fever. Diagnostic accuracy is evaluated only for differentiating viral-only adenovirus from adenovirus-bacterial co-infection within this pre-selected subgroup, and not in patients with suspected RTI or fever.
van Houten	2017	28012942	The study recruited children with fever of unknown origin or LRTI in EDs and pediatric wards but did not report the inpatient recruitment proportion or stratify results by ED vs. ward setting. It is unclear whether patient recruitment from the ED exceeded the 50% threshold specified in the eligibility criteria.
Wagner	2025	39249176	The study was conducted entirely among hospitalized inpatients and compared biomarker performance in patients with confirmed bloodstream infections versus those with confirmed viral infections. It did not include patients presenting with suspected RTI or undifferentiated fever in ED or urgent care settings.

## Appendix C. Risk of Bias Assessment Summaries for Non-DTA Studies

**Table A1:** Randomized controlled trial.

Cochrane Risk-of-Bias Tool for Randomized Trials (RoB 2.0)							
Study	Domains						
	1	2 (a)	2 (b)	3	4	5	Overall
Singer 2025 [19]	Low	Low	Low	Low	Low	Low	Low

**Table A2:** Non-randomized studies.

ROBINS-I Tool for Non-Randomized Studies of Interventions								
Study								
	1	2	3	4	5	6	7	Overall
Kalmovich 2023 [37]	S	M	L	M	L-M	M	M	S
Kalmovich 2025 [38]	S	M	L	M	L-M	M	M	S
Kalmovich 2026 [39]	S	S	L	M	M	M	M	S

L = low, M = moderate, S = serious.

**Table A3:** Cohort studies.

JBI Critical Appraisal Tool for the Assessment of Risk of Bias for Cohort Studies												
Study	Questions											
	1	2	3	4	5	6	7	8	9	10	11	Overall
Frohlich 2023 [36]	NA	NA	Yes	Unclear	Unclear	NA	Yes	NA	NA	NA	Yes	Moderate

NA = Not applicable.
